# 2,3-Dimethyl­phenyl benzoate

**DOI:** 10.1107/S160053680800963X

**Published:** 2008-04-16

**Authors:** B. Thimme Gowda, Sabine Foro, K. S. Babitha, Hartmut Fuess

**Affiliations:** a Department of Chemistry, Mangalore University, Mangalagangotri 574 199, Mangalore, India; b Institute of Materials Science, Darmstadt University of Technology, Petersenstrasse 23, D-64287 Darmstadt, Germany

## Abstract

The structure of the title compound (23DMPBA), C_15_H_14_O_2_, resembles those of phenyl benzoate (PBA), 3-methyl­phenyl benzoate (3MePBA), 2,6-dichloro­phenyl benzoate (26DC­PBA) and other aryl benzoates, with similar bond parameters. The dihedral angle between the benzene and benzoyl rings in 23DMPBA is 87.36 (6)°, compared with values of 55.7° in PBA, 79.61 (6)° in 3MePBA and 75.75 (10)° in 26DCPBA. The mol­ecules in 23DMPBA are packed into a chain-like structure in the direction of the *a* axis.

## Related literature

For related literature, see: Adams & Morsi (1976 ▶); Gowda *et al.* (2007*a* ▶,*b* ▶); Nayak & Gowda (2008 ▶).
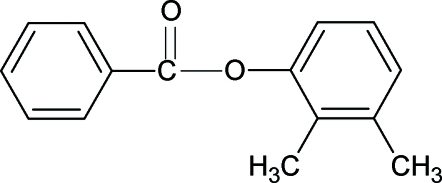


## Experimental

### 

#### Crystal data

C_15_H_14_O_2_
*M* *_r_* = 226.26
Monoclinic, 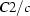

*a* = 15.190 (2) Å
*b* = 8.417 (1) Å
*c* = 20.604 (2) Å
β = 112.20 (1)°
*V* = 2439.0 (5) Å^3^
*Z* = 8
Cu *K*α radiation
μ = 0.65 mm^−1^
*T* = 299 (2) K
0.50 × 0.44 × 0.36 mm

#### Data collection

Enraf–Nonius CAD-4 diffractometer
Absorption correction: none
2328 measured reflections
2173 independent reflections
1886 reflections with *I* > 2σ(*I*)
*R*_int_ = 0.083
3 standard reflections frequency: 120 min intensity decay: none

#### Refinement

*R*[*F* ^2^ > 2σ(*F* ^2^)] = 0.048
*wR*(*F* ^2^) = 0.157
*S* = 1.07
2173 reflections
181 parameters
H atoms treated by a mixture of independent and constrained refinement
Δρ_max_ = 0.17 e Å^−3^
Δρ_min_ = −0.17 e Å^−3^

### 

Data collection: *CAD-4-PC* (Enraf–Nonius, 1996 ▶); cell refinement: *CAD-4-PC*; data reduction: *REDU4* (Stoe & Cie, 1987 ▶); program(s) used to solve structure: *SHELXS97* (Sheldrick, 2008 ▶); program(s) used to refine structure: *SHELXL97* (Sheldrick, 2008 ▶); molecular graphics: *PLATON* (Spek, 2003 ▶); software used to prepare material for publication: *SHELXL97*.

## Supplementary Material

Crystal structure: contains datablocks I, global. DOI: 10.1107/S160053680800963X/om2224sup1.cif

Structure factors: contains datablocks I. DOI: 10.1107/S160053680800963X/om2224Isup2.hkl

Additional supplementary materials:  crystallographic information; 3D view; checkCIF report

**Fig. 3.**
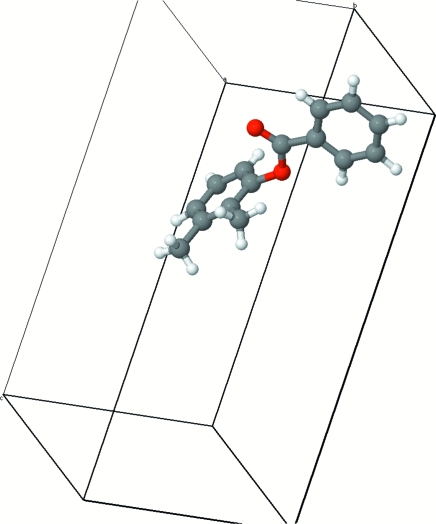
View of the molecule in the unit cell.

Enhanced figure: interactive version of Fig. 3

## Figures and Tables

**Fig. 1.**
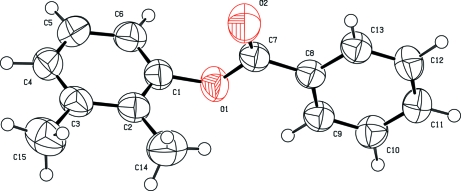
Molecular structure of the title compound, showing the atom labeling. Displacement ellipsoids are drawn at the 50% probability level.

**Fig. 2.**
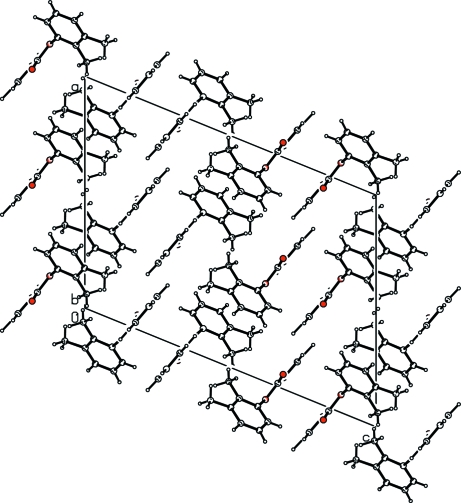
Molecular packing of the title compound as viewed down a axis.

## supplementary crystallographic information

### Comment

In the present work, as part of a study of the substituent effects on the structures of aryl benzoates (Gowda *et al.*, 2007*a*,*b*), the structure of 2,3-dimethylphenyl benzoate (23DMPBA) has been determined. The structure of 23DMPBA (Fig. 1) is similar to those of phenyl benzoate (PBA) (Adams & Morsi, 1976); 3-methylphenyl benzoate (3MePBA) (Gowda *et al.*, 2007*a*), 2,3-dichlorophenyl benzoate (23DCPBA), 2,6-dichlorophenyl benzoate (26DCPBA) and other aryl benzoates (Gowda *et al.*, 2007*b*). The bond parameters in 23DMPBA are similar to those in PBA, 3MePBA, 23DCPBA, 26DCPBA and other aryl benzoates. The dihedral angle between the benzene and benzoyl rings in 23DMPBA is 87.36 (6)°, compared to the values of 55.7° in PBA, 79.61 (6)° in 3MePBA and 75.75 (10)° in 26DCPBA. The molecules in the title compound are packed with the 2,3-dimethylphenyl and the benzoyl rings nearly orthogonal to each other, in the direction of the *a* axis (Fig. 2).

### Experimental

The title compound was prepared according to a literature method (Nayak & Gowda, 2008). The purity of the compound was checked by determining its melting point. It was characterized by recording its infrared and NMR spectra (Nayak & Gowda, 2008). Single crystals of the title compound were obtained by slow evaporation of an ethanolic solution.

### Refinement

The H atoms of the methyl groups were positioned with idealized geometry using a riding model with C—H = 0.96 Å The other H atoms were located in difference map, and their positional parameters were refined freely (C—H = 0.91 (2)–1.04 (2) Å). All H atoms were refined with isotropic displacement parameters (set to 1.2 times of the *U*_eq_ of the parent atom).

### Crystal data

**Table:**

C_15_H_14_O_2_	*F*_000_ = 960
*M**_r_* = 226.26	*D*_x_ = 1.232 Mg m^−^^3^
Monoclinic, *C*2/*c*	Cu *K*α radiation λ = 1.54180 Å
Hall symbol: -C 2yc	Cell parameters from 25 reflections
*a* = 15.190 (2) Å	θ = 6.1–21.6º
*b* = 8.417 (1) Å	µ = 0.65 mm^−^^1^
*c* = 20.604 (2) Å	*T* = 299 (2) K
β = 112.20 (1)º	Prism, colourless
*V* = 2439.0 (5) Å^3^	0.50 × 0.44 × 0.36 mm
*Z* = 8	

### Data collection

**Table:**

Enraf–Nonius CAD-4 diffractometer	*R*_int_ = 0.083
Radiation source: fine-focus sealed tube	θ_max_ = 66.9º
Monochromator: graphite	θ_min_ = 4.6º
*T* = 299(2) K	*h* = −18→1
ω/2θ scans	*k* = −10→0
Absorption correction: none	*l* = −23→24
2328 measured reflections	3 standard reflections
2173 independent reflections	every 120 min
1886 reflections with *I* > 2σ(*I*)	intensity decay: none

### Refinement

**Table:**

Refinement on *F*^2^	Hydrogen site location: inferred from neighbouring sites
Least-squares matrix: full	H atoms treated by a mixture of independent and constrained refinement
*R*[*F*^2^ > 2σ(*F*^2^)] = 0.048	*w* = 1/[σ^2^(*F*_o_^2^) + (0.0825*P*)^2^ + 1.2712*P*] where *P* = (*F*_o_^2^ + 2*F*_c_^2^)/3
*wR*(*F*^2^) = 0.157	(Δ/σ)_max_ = 0.037
*S* = 1.07	Δρ_max_ = 0.17 e Å^−^^3^
2173 reflections	Δρ_min_ = −0.17 e Å^−^^3^
181 parameters	Extinction correction: SHELXL97 (Sheldrick, 2008), Fc^*^=kFc[1+0.001xFc^2^λ^3^/sin(2θ)]^-1/4^
Primary atom site location: structure-invariant direct methods	Extinction coefficient: 0.0061 (5)
Secondary atom site location: difference Fourier map	

### Special details

**Table:**

Geometry. All e.s.d.'s (except the e.s.d. in the dihedral angle between two l.s. planes) are estimated using the full covariance matrix. The cell e.s.d.'s are taken into account individually in the estimation of e.s.d.'s in distances, angles and torsion angles; correlations between e.s.d.'s in cell parameters are only used when they are defined by crystal symmetry. An approximate (isotropic) treatment of cell e.s.d.'s is used for estimating e.s.d.'s involving l.s. planes.
Refinement. Refinement of *F*^2^ against ALL reflections. The weighted *R*-factor *wR* and goodness of fit *S* are based on *F*^2^, conventional *R*-factors *R* are based on *F*, with *F* set to zero for negative *F*^2^. The threshold expression of *F*^2^ > σ(*F*^2^) is used only for calculating *R*-factors(gt) *etc*. and is not relevant to the choice of reflections for refinement. *R*-factors based on *F*^2^ are statistically about twice as large as those based on *F*, and *R*-factors based on ALL data will be even larger.

### Fractional atomic coordinates and isotropic or equivalent isotropic displacement parameters (Å^2^)

**Table:**

	*x*	*y*	*z*	*U*_iso_*/*U*_eq_	
C1	0.11995 (13)	0.7351 (2)	0.40638 (10)	0.0564 (5)	
C2	0.11817 (13)	0.6814 (2)	0.46975 (10)	0.0595 (5)	
C3	0.17181 (15)	0.5454 (2)	0.49885 (10)	0.0642 (5)	
C4	0.22248 (16)	0.4726 (3)	0.46390 (12)	0.0704 (6)	
H4	0.2630 (18)	0.381 (3)	0.4882 (13)	0.085*	
C5	0.22058 (17)	0.5274 (3)	0.40080 (12)	0.0715 (6)	
H5	0.2588 (18)	0.475 (3)	0.3809 (14)	0.086*	
C6	0.16888 (15)	0.6605 (3)	0.37155 (11)	0.0649 (5)	
H6	0.1671 (17)	0.701 (3)	0.3293 (13)	0.078*	
C7	−0.01527 (13)	0.8854 (2)	0.33725 (10)	0.0567 (5)	
C8	−0.04911 (12)	1.0494 (2)	0.31758 (9)	0.0504 (4)	
C9	0.00808 (13)	1.1812 (2)	0.34477 (10)	0.0564 (5)	
H9	0.0723 (16)	1.168 (3)	0.3779 (11)	0.068*	
C10	−0.02700 (15)	1.3317 (2)	0.32619 (11)	0.0623 (5)	
H10	0.0154 (16)	1.430 (3)	0.3487 (12)	0.075*	
C11	−0.11960 (15)	1.3535 (3)	0.28000 (11)	0.0631 (5)	
H11	−0.1449 (17)	1.458 (3)	0.2661 (13)	0.076*	
C12	−0.17667 (14)	1.2230 (3)	0.25200 (11)	0.0645 (5)	
H12	−0.2430 (17)	1.236 (3)	0.2159 (13)	0.077*	
C13	−0.14166 (13)	1.0727 (3)	0.27052 (11)	0.0591 (5)	
H13	−0.1795 (16)	0.987 (3)	0.2533 (12)	0.071*	
C14	0.06335 (18)	0.7675 (4)	0.50556 (15)	0.0871 (8)	
H14A	0.0134	0.7002	0.5076	0.105*	
H14B	0.0362	0.8622	0.4798	0.105*	
H14C	0.1051	0.7954	0.5522	0.105*	
C15	0.1773 (2)	0.4813 (3)	0.56843 (13)	0.0935 (8)	
H15A	0.1143	0.4629	0.5670	0.112*	
H15B	0.2091	0.5568	0.6046	0.112*	
H15C	0.2121	0.3831	0.5780	0.112*	
O1	0.07702 (9)	0.88173 (15)	0.37981 (8)	0.0675 (4)	
O2	−0.06258 (11)	0.76695 (18)	0.31914 (10)	0.0883 (6)	

### Atomic displacement parameters (Å^2^)

**Table:**

	*U*^11^	*U*^22^	*U*^33^	*U*^12^	*U*^13^	*U*^23^
C1	0.0502 (9)	0.0455 (9)	0.0609 (10)	−0.0046 (7)	0.0065 (8)	−0.0017 (8)
C2	0.0535 (10)	0.0564 (11)	0.0610 (11)	−0.0095 (8)	0.0129 (8)	−0.0072 (8)
C3	0.0677 (11)	0.0563 (11)	0.0564 (10)	−0.0138 (9)	0.0095 (9)	0.0000 (8)
C4	0.0738 (13)	0.0501 (11)	0.0703 (13)	0.0013 (10)	0.0079 (10)	−0.0009 (9)
C5	0.0752 (13)	0.0619 (12)	0.0719 (13)	0.0045 (10)	0.0217 (11)	−0.0120 (10)
C6	0.0691 (12)	0.0617 (12)	0.0573 (11)	−0.0039 (9)	0.0164 (9)	−0.0044 (9)
C7	0.0490 (9)	0.0546 (11)	0.0584 (10)	−0.0054 (8)	0.0112 (8)	−0.0043 (8)
C8	0.0471 (9)	0.0549 (10)	0.0493 (9)	−0.0032 (7)	0.0182 (7)	−0.0015 (7)
C9	0.0490 (9)	0.0566 (11)	0.0596 (10)	−0.0050 (8)	0.0158 (8)	−0.0020 (8)
C10	0.0629 (11)	0.0524 (11)	0.0707 (12)	−0.0060 (9)	0.0244 (9)	−0.0003 (9)
C11	0.0648 (12)	0.0569 (11)	0.0691 (12)	0.0077 (9)	0.0271 (10)	0.0086 (9)
C12	0.0511 (10)	0.0692 (12)	0.0668 (12)	0.0066 (9)	0.0148 (9)	0.0069 (9)
C13	0.0471 (9)	0.0610 (11)	0.0645 (11)	−0.0054 (8)	0.0157 (8)	−0.0020 (9)
C14	0.0745 (14)	0.0993 (19)	0.0908 (17)	−0.0057 (13)	0.0348 (13)	−0.0173 (14)
C15	0.111 (2)	0.0891 (17)	0.0706 (14)	−0.0173 (15)	0.0230 (13)	0.0152 (13)
O1	0.0532 (7)	0.0483 (8)	0.0808 (9)	−0.0021 (5)	0.0025 (6)	0.0035 (6)
O2	0.0634 (9)	0.0556 (9)	0.1143 (13)	−0.0099 (7)	−0.0022 (8)	−0.0049 (8)

### Geometric parameters (Å, °)

**Table:**

C1—C6	1.365 (3)	C8—C9	1.390 (2)
C1—C2	1.391 (3)	C9—C10	1.372 (3)
C1—O1	1.407 (2)	C9—H9	0.96 (2)
C2—C3	1.402 (3)	C10—C11	1.381 (3)
C2—C14	1.492 (3)	C10—H10	1.04 (2)
C3—C4	1.380 (3)	C11—C12	1.383 (3)
C3—C15	1.504 (3)	C11—H11	0.96 (2)
C4—C5	1.369 (3)	C12—C13	1.370 (3)
C4—H4	1.00 (3)	C12—H12	1.01 (3)
C5—C6	1.370 (3)	C13—H13	0.91 (2)
C5—H5	0.94 (3)	C14—H14A	0.9600
C6—H6	0.92 (2)	C14—H14B	0.9600
C7—O2	1.203 (2)	C14—H14C	0.9600
C7—O1	1.344 (2)	C15—H15A	0.9600
C7—C8	1.475 (3)	C15—H15B	0.9600
C8—C13	1.387 (3)	C15—H15C	0.9600
			
C6—C1—C2	123.46 (19)	C8—C9—H9	120.3 (14)
C6—C1—O1	117.59 (18)	C9—C10—C11	120.18 (19)
C2—C1—O1	118.66 (18)	C9—C10—H10	119.9 (13)
C1—C2—C3	116.89 (19)	C11—C10—H10	119.9 (13)
C1—C2—C14	121.2 (2)	C10—C11—C12	119.76 (19)
C3—C2—C14	121.9 (2)	C10—C11—H11	121.1 (15)
C4—C3—C2	119.20 (19)	C12—C11—H11	119.1 (15)
C4—C3—C15	119.8 (2)	C13—C12—C11	120.09 (18)
C2—C3—C15	121.0 (2)	C13—C12—H12	118.8 (14)
C5—C4—C3	122.0 (2)	C11—C12—H12	121.0 (14)
C5—C4—H4	121.7 (14)	C12—C13—C8	120.62 (19)
C3—C4—H4	116.3 (14)	C12—C13—H13	120.3 (14)
C4—C5—C6	119.7 (2)	C8—C13—H13	119.0 (15)
C4—C5—H5	117.5 (16)	C2—C14—H14A	109.5
C6—C5—H5	122.7 (16)	C2—C14—H14B	109.5
C1—C6—C5	118.7 (2)	H14A—C14—H14B	109.5
C1—C6—H6	120.0 (15)	C2—C14—H14C	109.5
C5—C6—H6	121.3 (15)	H14A—C14—H14C	109.5
O2—C7—O1	122.50 (17)	H14B—C14—H14C	109.5
O2—C7—C8	125.80 (16)	C3—C15—H15A	109.5
O1—C7—C8	111.70 (15)	C3—C15—H15B	109.5
C13—C8—C9	118.88 (18)	H15A—C15—H15B	109.5
C13—C8—C7	118.69 (16)	C3—C15—H15C	109.5
C9—C8—C7	122.43 (16)	H15A—C15—H15C	109.5
C10—C9—C8	120.46 (17)	H15B—C15—H15C	109.5
C10—C9—H9	119.2 (14)	C7—O1—C1	119.33 (14)
			
C6—C1—C2—C3	1.5 (3)	O1—C7—C8—C13	176.36 (16)
O1—C1—C2—C3	−172.15 (15)	O2—C7—C8—C9	175.1 (2)
C6—C1—C2—C14	−179.93 (19)	O1—C7—C8—C9	−4.1 (3)
O1—C1—C2—C14	6.5 (3)	C13—C8—C9—C10	1.0 (3)
C1—C2—C3—C4	−0.2 (3)	C7—C8—C9—C10	−178.57 (18)
C14—C2—C3—C4	−178.79 (19)	C8—C9—C10—C11	−0.2 (3)
C1—C2—C3—C15	177.84 (19)	C9—C10—C11—C12	−0.6 (3)
C14—C2—C3—C15	−0.8 (3)	C10—C11—C12—C13	0.6 (3)
C2—C3—C4—C5	−1.3 (3)	C11—C12—C13—C8	0.3 (3)
C15—C3—C4—C5	−179.3 (2)	C9—C8—C13—C12	−1.0 (3)
C3—C4—C5—C6	1.5 (3)	C7—C8—C13—C12	178.55 (18)
C2—C1—C6—C5	−1.2 (3)	O2—C7—O1—C1	−2.1 (3)
O1—C1—C6—C5	172.43 (17)	C8—C7—O1—C1	177.08 (16)
C4—C5—C6—C1	−0.3 (3)	C6—C1—O1—C7	96.1 (2)
O2—C7—C8—C13	−4.5 (3)	C2—C1—O1—C7	−90.0 (2)
